# Monitoring for a new *I3* resistance gene-breaking race of *F. oxysporum* f. sp. *lycopersici* (Fusarium wilt) in California processing tomatoes following recent widespread adoption of resistant (F3) cultivars: Challenges with race 3 and 4 differentiation methods

**DOI:** 10.3389/fpls.2023.1088044

**Published:** 2023-03-31

**Authors:** Cassandra L. Swett, Johanna Del Castillo Múnera, Elizabeth Hellman, Erin Helpio, Megan Gastelum, Elver Lopez Raymundo, Heather Johnson, Rino Oguchi, Aimee Hopkins, Justine Beaulieu, Fernando Rodriguez

**Affiliations:** Swett Lab, Department of Plant Pathology, University of California, Davis, CA, United States

**Keywords:** diagnostics, phenotyping, race emergence, *Fusarium* wilt, tomato, overcoming resistance

## Abstract

Fusarium wilt, caused by *Fusarium oxysporum* f. sp. *lycopersici* (Fol), causes losses in tomato production worldwide, with major impacts on Californian tomato processing. Single-gene resistance is the primary management tool, but its efficacy has been compromised following the emergence of two successive resistance-breaking races, which, in California, emerged within 12 years of resistance deployment. Fol race 3-resistant (F3) processing tomato cultivars (containing the *I3* resistance gene) were deployed in the state starting in approximately 2009. The emergence of a new resistance-breaking race (which would be called race 4) is imminent, and early detection will be critical to delay the spread while new resistance is sought. The detection of Fol race 4 is challenged by the lack of validated, rapid, and accurate diagnostic tools. In evaluating *in planta* phenotyping methods, this study found that rapid seedling phenotyping is not reliable and generates false positives for nonpathogens. Longer (10 weeks) mature plant assays are the most reliable, but may not be sufficiently timely. As an additional challenge, based on field and greenhouse studies, Fol race 3 can cause symptoms in resistant F3 cultivars at frequencies greater (30%) than expected for off-types (<2%). We developed a three-F3 cultivar *in planta* assay to overcome the challenges this posed to differentiating Fol race 3 and Fol race 4. Using the assay, we determined that all putative resistance-breaking cases were Fol race 3; Fol race 4 was not detected in these early survey efforts. These results highlight the need for developing rapid Fol race 4 detection tools and a better understanding of the factors underlying inconsistent *I3* gene expression in Fol race 3.

## Introduction

Documented in over 30 countries, Fusarium wilt, which is caused by *Fusarium oxysporum* f. sp. *lycopersici* (Fol), is a major driver of yield losses in tomato and has significant impacts in California, one of the primary global producers of processing tomatoes (Cai et al., 2003; McGovern, 2015; Fisher, 2017). Tomato cultivars with single-gene resistance have been the keystone of integrated disease management (McGovern, 2015; Chitwood-Brown et al., 2021a). However, resistance efficacy has been compromised by the emergence of two successive resistance-breaking races: race 2 and race 3 (Di Pietro et al., 2003; Takken and Rep, 2010; McGovern, 2015).

Fusarium wilt of tomato (later designated Fol race 1) was first described in the UK by Massee, 1895, although the first description aligning with a modern concept of Fusarium wilt was in the USA (Florida) in 1899 (Smith, 1899). Fusarium wilt (race 1) spread rapidly around the globe and was detected in California in the late 1940s (Hannah, 1954; Walker, 1971). The first resistance gene (*I1*) was discovered in 1939 by Bohn and Tucker, 1939. In California, resistance was deployed in processing tomatoes around 1959, about 15 years after the first detection of Fusarium wilt in the state (Hanna et al., 1964).

The *I1*-resistance-breaking Fol (race 2) was first detected in the USA, in Ohio in 1945 (Alexander, 1945; Walker, 1971) and again in California in 1970 (San Joaquin County) (Zobel, 1971), representing about 11 years of resistance gene efficacy in the state (1959–1970). Fol race 2 resistance (*I2* gene) was developed between 1955 and 1965 (Alexander and Hoover, 1955; Alexander, 1959; Stall and Walter, 1965). Materials with race 2 resistance (F2 cultivars) were first commercially available by 1969 (Strobel et al., 1969; Gabe, 1975). Their use began in California around 1975–1977 (Thomas 1979), with F2 materials commonly used by 1980, 10 years after race 2 had been detected in the state (Miyao, 1980; Miyao and Debiase, 1996).

The *I2*-resistance-breaking Fol race 3 (Fol R3) was first detected in Australia in 1978 (Grattidge and O’Brien, 1982). It was first reported in the USA, in California, in 1987 (Davis et al., 1988; Cai et al., 2003), representing up to 12 years of resistant cultivar efficacy (1975–1987) and only 7 years when dating from the widespread adoption of the F2 cultivar. Fol R3-resistant (F3) materials (*I3* gene) were in development starting in the early 1980s (Jones and Crill, 1974; McGrath and Maltby, 1988; Huang and Lindhout, 1997). In California, the use of the F3 cultivar (carrying the *I1*, *I2*, and *I3* resistance genes) began around 2009 (22 years after detection), with widespread use starting in about 2016, with 10% of the northern San Joaquin Valley and 20% of Sutter County (northern counties) planting F3 cultivars (PTAB, 2016). The adoption of the F3 cultivar has been hindered in this and in other regions due, in part, to poor yield and performance traits, which significantly improved starting around 2019.

A new resistance-breaking Fol race (which would be called race 4, as the fourth successive race) has not been documented anywhere in the world to date. If the previous timeline provides any indication of future resistance emergence patterns, we would expect the emergence of the new resistance-breaking race 4 between 2021 (12 years from the initial use of the F3 cultivar in 2009) and 2023 (7 years from widespread use in 2016).

Early detection of race 4 will be critical to manage infested sources and to mitigate its spread in order to preserve the efficacy of the F3 cultivar while breeders work to develop race 4-resistant lines. While history has shown a rapid turnaround between the detection of the new race and the identification of the resistance gene (≤10 years), the time to commercial adoption can be over 20 years. The rapid advancement of resistant materials is hindered by the process of identifying and eliminating linkage drag issues and backcrossing to reincorporating traits required for a commercially competitive product (Chitwood-Brown et al., 2021b). Early detection and containment of race 4 will therefore be critical to preserving the efficacy of the existing resistance while new resistant materials are developed and deployed. As California (and particularly the Sacramento Valley) is thought to be a site of origin for race emergence (Cai et al., 2003), it is one of the primary regions under aggressive race 4 monitoring worldwide. The year 2019 was the first year when an F3 cultivar was the most widely grown cultivar in the state (PTAB, 2019)—this widespread adoption provided the opportunity to test for the emergence of Fol race 4 across the state.

The detection and rapid response to the emergence of resistance-breaking race 4 depend on accurate and timely diagnosis. Field diagnosis, while rapid, is hindered by the diversity of pathogens that cause Fusarium wilt-like symptoms in F3 cultivars, including other *Fusarium* species such as *Fusarium oxysporum* f. sp. *racidis lycopersici* (Forl; the cause of Fusarium crown and root rot) and pathogens in the *Fusarium solani* species complex (Romberg and Davis, 2007; Paugh et al., 2022). Traditional laboratory diagnosis of Fol using morphology-based methods can provide results quickly, but morphology cannot be used to accurately identify species or to provide any indication of formae specialis or race identity. Molecular-based methods offer a promising means for both accurate and timely diagnosis (e.g., Hirano and Arie, 2006; Lievens et al., 2008; Ayukawa et al., 2016; Carmona et al., 2020). However, thus far, efforts to validate these (including with California isolates) (Swett, unpublished) have revealed a lack of specificity, as highlighted by Jelinski et al. (2017).

Due to these challenges, *in planta*-based methods continue to provide the standard for the accurate differentiation of pathogenic from nonpathogenic strains, segregation of the different formae specialis, and differentiation of Fol races (e.g., López-Benítez et al., 2018; Cabral et al., 2020; Munawar et al., 2020; Armenta-López et al., 2021; de Oliveira et al., 2021). *In planta* identification methods vary in terms of plant age, inoculation method, and the duration needed for symptom development, with results in 2–12 weeks, depending on the method. There has been no rigorous comparative analysis of the accuracy and efficacy of these different methods to achieve race 4-oriented diagnostic goals. Of particular concern is that the rapid seedling-based methods, while appealing in terms of speed and ease, have not been evaluated for the risk of generating false positives for the nonpathogenic strains of *F. oxysporum* (which may appear to cause disease in seedlings).

*In planta* diagnostic methods rely on the durability of the resistance gene to withstand the target race. Preliminary monitoring efforts for Fol race 4 have indicated that the *I3* resistance gene in processing tomatoes is inconsistently expressed—a phenomenon which may allow Fol R3 to cause symptoms in resistant materials, generating false positives for race 4. This has the potential to erode the utility of using differential lines for the identification of Fol race 4 *in planta*. However, it is also known that there is a certain allowable percentage of “off-type” seeds (~2%) that failed to hybridize and do not contain the *I3* gene. In a highly infested field, this could allow Fusarium wilt development in up to ~2% of the field. Studies are needed to better understand this phenomenon and overcome the challenges it poses to both *in planta* diagnostics and the use of resistant cultivars as a management tool.

In support of Fol race 4 monitoring goals, the specific objectives of these study were to: 1) evaluate *in planta* tools for speed and accuracy in differentiating pathogenic and nonpathogenic *F. oxysporum* subspecies and Fol races in order to develop a standardized protocol applicable to the identification of race 4; 2) examine the relationship between Fol R3 and F3 cultivars to determine whether Fol R3 is causing disease in F3 materials; 3) use this information to develop a standardized, accurate phenotyping method for Fol race 4 monitoring; and 4) conduct statewide surveys in California processing tomatoes (2017–2021) to assess whether Fol race 4 is detectable in the state 8–12 years after the initial F3 cultivar deployment, 1–5 years after its widespread use in affected regions.

## Materials and methods

### Evaluating *in planta* tools for accuracy and speed to develop a standardized method for Fol race 4 identification

To provide a standardized protocol for the diagnosis of Fol race 4 that optimized accuracy and duration, we undertook to evaluate *in planta* diagnostics tools representing three categories: *in vitro* seedling assay (short time frame), young mature plant high-throughput seeded tray assay (medium time frame), and young mature potted plant assay (longer time frame). These were evaluated for their ability to differentiate Fol from Forl, to differentiate the pathogenic and nonpathogenic *F. oxysporum* strains present in tomatoes, and to accurately identify Fol R3 (allowing differentiation from race 4). The isolates evaluated in all *in planta* methods consisted of: 1) *F. oxysporum* f. sp. *lycopersici* race 3 (Fol R3) isolate CS3; 2) *F. oxysporum radicis lycopersici* (Forl) isolate CS141; and 3) the nonpathogenic *F. oxysporum* (Fonp) isolate CS351, which was originally recovered from tomato and previously characterized as unable to cause disease. Mock-inoculated environmental controls consisted of 0.1% water agar or untreated plants, as described below.

#### Inoculum preparation

The inoculum was prepared by streaking colonized filter papers across full-strength potato dextrose agar (PDA) in 10-cm diameter plates and incubating for 7 days under constant fluorescent light at room temperature (24°CC). The spore suspension was prepared by adding sterile 0.6% KCl into 7-day-old active growing mycelia of each isolate and filtered through two layers of sterile cheesecloth. The spore densities were quantified using a hemacytometer and adjusted to 10^6^ spores/ml by the addition of 0.1% water agar (Swett et al., 2016).

#### Plant material

All trials used processing tomato varieties, with the exception that, in some cases, the heirloom tomato cv. Brandywine was used as a positive control since it was more easily obtainable. To stimulate even germination, *Solanum lycopersicum* seeds (see **Table 1** for cultivars) were soaked in 70% ethanol for 10 min, 1% sodium hypochlorite for 10 min, and then rinsed with distilled water and air dried (Del Castillo Múnera et al., 2019). Within 24 h of treatment, the seeds were planted according to the different methods evaluated below.

**Table 1:** Tomato cultivars used in all studies.

Cultivar (R)^a^	Company	Resistant to	Study^b^
EP 7 or Brandywine (none)	NA	None	Plant tray assay, greenhouse F3, Fol race 4 monitoring (Brandywine)
H 9036 (F1)	Heinz	Fol race 1	Plant tray assay, greenhouse F3, Fol race 4 monitoring
H 8504 (F2)	Heinz	Fol race 1, race 2	Lab bioassay, single pot, plant tray assay, field F3, greenhouse F3, Fol race 4 monitoring
HM 58841 (F2)	HM Clause	Fol race 1, race 2	Lab bioassay, single pot, plant tray assay
H 1310 (F3)	Heinz	Fol race 1, race 2, race 3	Lab bioassay, single pot, plant tray assay, field F3
N 6428 (F3)	Nunhems	Fol race 1, race 2, race 3	Plant tray assay, field F3, greenhouse F3, Fol race 4 monitoring
HM 58801 (F3)	HM Clause	Fol race 1, race 2, race 3	Field, greenhouse F3, monitoring
HMX 4909 (Fr)	HM Clause	Forl	Lab bioassay, single pot, plant tray assay, Fol race 4 monitoring

Fol, Fusarium oxysporum f. sp. lycopersici; Forl, Fusarium oxysporum radicis lycopersici.

a Resistance abbreviation used in text: None, no resistance; F1, resistant to Fol race 1 only; F2, resistant to Fol race 2; F3, resistant to Fol race 3; Fr, resistant to Forl.

b Comparative phenotyping studies: lab bioassay, high-throughput (tray) assay, single-pot assay. F3 durability studies: greenhouse F3 and field F3. Fol race 4 monitoring: statewide Fol race 4 monitoring over 5 years. NA, Not Apply.

#### Lab bioassay

##### Experimental design and treatment implementation

The primary purpose of this study was to determine the feasibility of using this quick assay method (21 days in total) to accurately separate Fol from Forl and Fonp strains using differential cultivar susceptibility/resistance profiles. As preliminary studies had indicated a high rate of false-positive results for every Fol race, we only evaluated one Fol race (race 3) as a case study, including only those cultivars needed to identify this race and Forl.

The protocol was adapted from a protocol provided by a local private seed company, which served as their standard rapid test for segregating pathogenic and nonpathogenic *F. oxysporum* strains. The experiment was arranged as a completely randomized design, with experimental units consisting of three sterile magenta boxes (7.5 cm × 6 cm × 6 cm), each containing 10 seeds of each cultivar (i.e., H 8504, HM 58841, HMX 4909, and H 1310) (**Table 1**), for each pathogen treatment (i.e., Fol R3, Forl, Fonp, and mock-inoculated). The experiment was conducted two times.

Magenta boxes were filled (up to 3 cm from the bottom) with 5% water agar, and once water agar was solidified, a 55-mm filter paper (Whatman, Maidstone, UK) was placed on top and moistened with 1 ml sterile deionized water. Of the same cultivar per box, 10 seeds were placed equidistance (at ~10 mm spacing) on top of the filter paper, and the magenta boxes were closed and randomly placed on a laboratory shelf under constant fluorescent light at 24°CC. After 15 days, germinating (15-day-old) seedlings at the cotyledonary leaf stage were inoculated by decanting 1 ml of the corresponding spore suspension (10^6^ spores/ml of Fol, Forl, or Fonp) or sterile 0.1% water agar (mock inoculation) over the filter paper. The inoculated plants were maintained under constant fluorescence light at 24°CC for 6 days. Differential cultivar response was then quantified based on the number of seedlings per magenta box with hypocotyl lesions and extensive necrosis (dead or dying).

##### Statistical analyses

**Figure 1:**
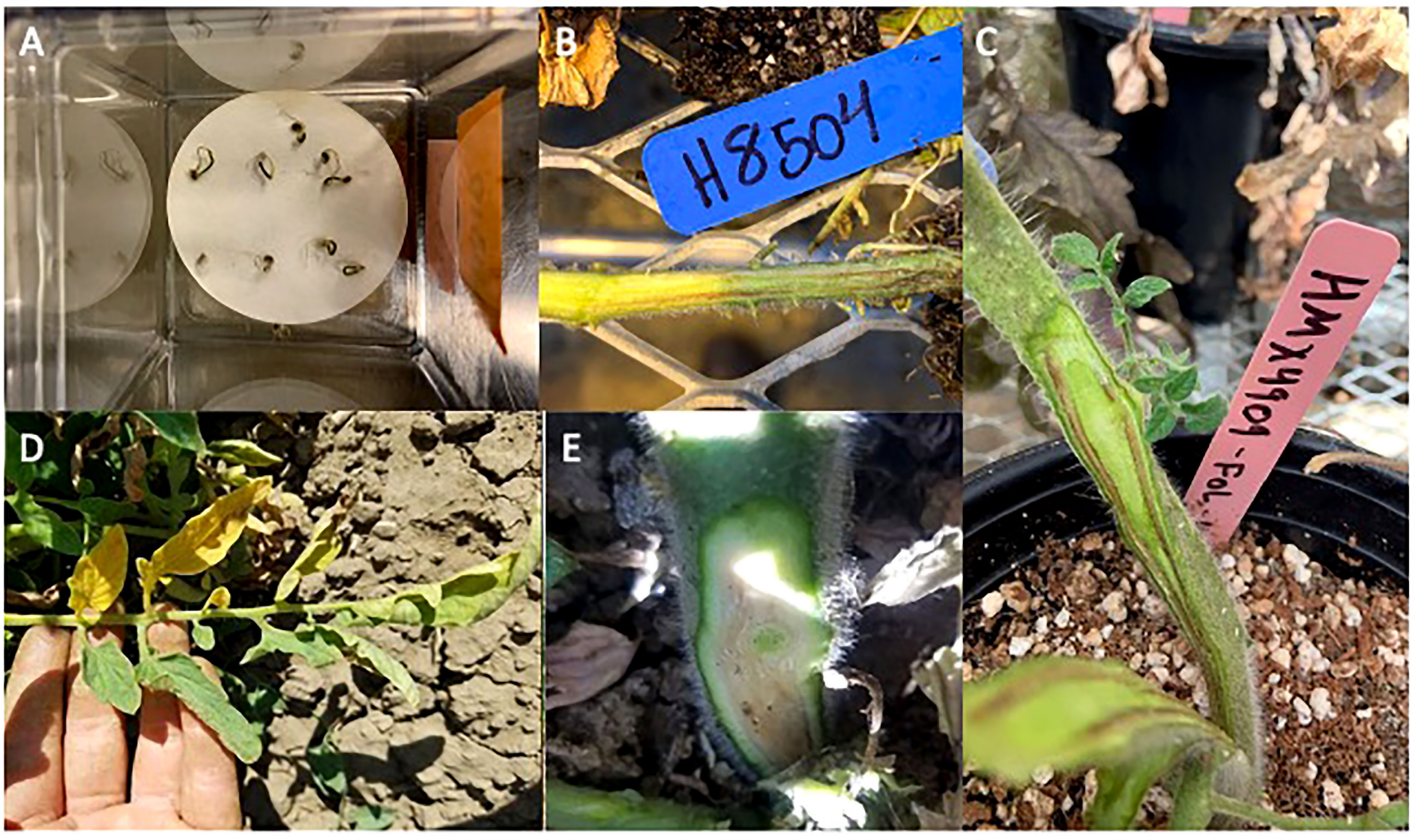
Symptom development of tomato at different stages inoculated with *Fusarium oxysporum* (f) sp. *lycopersici* race 3 (Fol R3). **(A)** Seedling decline and H 1310 seedling mortality from the laboratory bioassay. **(B)** Vascular discoloration of H 8504 developed in a high-throughput assay. **(C)** Vascular discoloration of HMX 4909 developed in a single-pot assay. **(D)** One-sided leaf chlorosis observed in a Fol R3 infested field. **(E)** Vascular discoloration observed in an F3 cultivar under Fol R3 pressure in the field trial.

Experiment replicates were considered random variables. Pathogen and cultivar treatment were considered fixed variables. The pathogen × cultivar treatment and experiment replicate treatment interactions were not significant (*p* = 0.248 and 0.469, respectively); therefore, all data were analyzed together. A replicate unit consisted of a single treated magenta box containing 10 seedlings, for a total of three replicates per treatment per experiment and six total replicates/treatment across experiments. The incidence (percentage of seedlings/box) of hypocotyl and radicle lesions and the incidence of seedling mortality (percentage of seedings/box dying or dead) (**Figure 1A**) were analyzed utilizing analysis of variance (ANOVA) with a type II test, which used R version 4.0 (R foundation for Statistical Computing, Vienna, Austria) with packages “car” and “lm4.” The percentage data were arcsin square root transformed before the analyses. If ANOVA was significant for the main effects, the treatment means were compared using *post-hoc* Tukey’s analysis test at *α* = 0.05.

#### Young plant tray assay

##### Experimental design and treatment implementation

The primary purpose of this study was aligned with the above laboratory bioassay, with the additional goal of evaluating the efficacy of using differential cultivars to separate all races as preliminary studies had indicated low to no false-positive rates for all races. Thus, we included cultivars that differentiated all races using replicate resistant cultivars when commercially available. This study was initiated with seedlings at the primary leaf stage grown in trays containing a potting mix and ran for a longer duration (plants inoculated at 2 weeks old and kept in the greenhouse for 7–9 weeks). The experiment was conducted in the UC Davis core greenhouse complex and was arranged as a split-plot design on a single bench. Each plot consisted of a 128-plug tray containing all seven differential cultivars (i.e., Brandywine, H 9036, H 8504, HM 58841, H 1310, N 6428, and HMX 4909) (**Table 1**). Each tray (plot) was inoculated with a different pathogen treatment (i.e., Fol R3, Forl, Fonp, and mock-inoculated), with one tray per pathogen treatment. The experiment was conducted twice.

The trays were filled with Sungrow propagation mix #4 (Agawam, MA, USA) fertilized with osmocote (2.2 ml/L). Two seeds from each cultivar were sown in 16 plugs of a 128-plug tray (two rows per tray) and thinned to one per plug shortly after emergence. Trays containing 2-week-old seedlings at the early primary leaf stage were submerged up to the seedling crown (just above the potting mix surface) in a sterilized plastic bin containing 2 L of spore suspension of each *F. oxysporum* treatment for 2 min. The trays were then drained and inserted into a solid-bottom plastic tray. The mock treatment tray was left untreated. Experiments 1 and 2 ran for 7 and 9 weeks, respectively (the latter adjustment was made as symptoms were not as apparent at 7 weeks), from February to April of 2020 (experiment 1) and 2021 (experiment 2) [24.0°CC ± 8°CC, 16:8 h light/dark (L/D)]. The trays were bottom watered daily and fertilized every other week with a mix of N (150 ppm)/K (200 ppm)/P (50 ppm)/Ca (175 ppm)/S (120 ppm).

Symptom development was monitored weekly. At the end of the experiment, each plant was first evaluated for the presence/absence of decline (wilting/loss of turgor in young leaves). The stem was then cut longitudinally starting at the soil line to evaluate the presence/absence of crown rot (Forl) or vascular discoloration (Fol) (**Figure 1B**). Disease incidence was calculated as the percentage of plants (out of 16) within each tray that showed decline and crown rot/vascular discoloration.

##### Statistical analyses

Experiment replicates were considered random variables. Pathogen and cultivar treatment were considered fixed variables. The pathogen × cultivar treatment interaction was significant (*p* < 0.01); therefore, cultivar differences were analyzed separately by pathogen treatment. A replicate unit consisted of a single tray containing 16 plants per cultivar × pathogen treatment; across experiments, each treatment was replicated twice. Incidences of vascular discoloration/crown rot were analyzed with ANOVA using a type II test (“car” package in R). The percentage data were arcsin square root transformed before the analyses. If ANOVA was significant for the main effects, the treatment means were compared using *post-hoc* Tukey’s analysis test at *α* = 0.05. In some cases, the canopy symptom data were more variable across experiments, preventing experiment combination—these results were represented by the presence/absence data as an indication of the efficacy of differential cultivar use. Otherwise, ANOVA and comparisons of the means were conducted as above.

#### Young plant single-pot assay

##### Experimental design and treatment implementation

The primary purpose of this study was aligned with the above *in planta* tray assays. Due to space limitations, in this study, only one replicate resistant cultivar was included for each pathogen. The study was initiated with seedlings at the primary leaf stage (plants inoculated at 3 weeks old). Plants were grown and inoculated in individual pots, and the experiment ran for 10–11 weeks, the longest duration. The experiment was conducted on raised benches in the UC Davis CORE greenhouse complex, arranged on a single bench in a split-plot design, with pathogen treatment (i.e., Fol R3, Forl, Fonp, and mock-inoculated) as the main plot. Each main plot contained six plants per cultivar (i.e., H 8504, HM 58841, HMX 4909, and H 1310), which were randomly arranged (**Table 1**), and each pot was inoculated with a different pathogen treatment (i.e., Fol R3, Forl, Fonp, or mock-inoculated). The experiment was conducted twice.

The seeds of each cultivar were sown on 3.78-L pots containing UC agronomy mix (equal parts sand, redwood sawdust, sphagnum peat moss, and pumice rock mixed with 1% dolomite lime). Three-week-old plants were inoculated by decanting 50 ml of the corresponding spore suspension on the plant crown. For the mock inoculation, 50 ml of 0.1% water agar was added. Experiment 1 was conducted from February to April 2020 (10 weeks), while experiment 2 was conducted from September to December 2020 (11 weeks), with adjustments in duration reflecting the differences in time to symptom development between trials (as above). Plants were watered daily and fertilized once a week with the rates specified above (24.0°CC ± 8°CC, 16:8 h L/D), and symptom development was monitored weekly. At 10–11 weeks, the plant canopy was assessed for the presence/absence of canopy decline (wilt/canopy collapse), the plants were removed from the pots, and the crowns and stems were cut in cross-sections and longitudinally to evaluate tissue necrosis. Disease incidence was quantified based on the percentage of plants (out of six) with vascular discoloration or crown rot (**Figure 1C**) and canopy decline.

##### Statistical analyses

Experiment replicates were considered random variables. Pathogen and cultivar treatment were considered fixed variables. The pathogen × cultivar treatment interaction was significant (*p* < 0.01); therefore, data were analyzed separately by pathogen treatment. Each plot was treated as a replicate unit (*n* = 2). The response variables within pathogen treatment were not significantly different among the experiment replicates; hence, the data were pooled and analyzed together. Incidences of vascular discoloration/crown rot and decline (wilting/loss of turgor in mature plants) were analyzed with ANOVA using a type II test (“car” package in R). Percentage data were arcsin square root transformed before the analyses. When ANOVA was significant for the main effects, the treatment means were compared using *post-hoc* Tukey’s analysis test at *α* = 0.05.

### Evaluating the relationship between Fol R3 and F3 processing tomato cultivars to overcome the challenges of *in planta* diagnostics

The overall goal of this study was to document the phenomenon of Fol R3-driven disease development in F3 processing tomato cultivars, as well as to overcome the challenges this phenomenon poses to *in planta* diagnostics. To this end, we conducted a 2-year trial in a Fol R3-infested experimental field to evaluate three F3 cultivars for resistance gene durability by assessing Fusarium wilt development over the field season. We coupled this with greenhouse trials evaluating the durability of three F3 cultivars against Fol R3. As molecular testing tools were not available to confirm the presence of the *I3* resistance gene in individual plants (due to the proprietary nature of the resistance gene region in the private sector), we utilized a threshold-based analysis, which referenced the 2% threshold for allowable off-type seeds (failed hybridization, leading to the absence of the *I3* gene). Based on this, Fusarium wilt development in ≤2% of plants was used to indicate a likely role of off-types (plants without the *I3* gene) in allowing symptom manifestation, whereas Fusarium wilt development in >2% of plants was used as an indicator that the *I3* gene was present in at least some affected plants, but may not have been effectively expressed, thus allowing Fol R3 to cause disease.

These studies were utilized to develop a rigorous three-F3 cultivar greenhouse phenotyping method that could overcome potential problems in *I3* resistance gene expression and allow accurate identification of Fol race 4 in processing tomatoes. This method was employed in a 5-year survey to determine whether a resistance-breaking race was detectable in commercial F3 processing tomato fields in California.

#### Field-based evaluation of the relationship between the Fol R3 and F3 cultivars

##### Experimental design and treatment implementation

We examined three Fol race 3-resistant (F3 cultivars) processing tomato cultivars, each representing a different industry source: N 6428 (Nunhems), H 1310 (Heinz), and HM 58801 (HM Clause). We included the Fol race 2 (F2) susceptible cultivar H 8504 as a positive control (**Table 1**). Clean seed material was provided by the breeding companies, and the seeds were further treated with 1% sodium hypochlorite, as described above, prior to planting in order to minimize the presence of any contaminant pathogens. This study was conducted at the UC Davis Armstrong Plant Pathology Research Station (GPS: 38.522426, −121.757284; soil type: Yolo clay loam) from May to September of 2018 and was repeated during the same months in 2019. The study was arranged as a split-plot randomized complete block design, in which pathogen treatment (non-inoculated or inoculated) was treated as the main plot. The non-infested control was included as a check for Fusarium wilt-mimicking symptoms. The cultivars were randomly allocated to single 4.5-m plots within each of three blocks (rows), with 30–35 cm plant spacing for a total of ~15 plants per cultivar per block. Each field plot was surrounded by two buffer rows in 2019, but not in 2018.

Infested and non-infested field plots were located in the middle of the experiment station, situated approximately 200 m from each other to avoid cross-contamination. The non-infested control field was prepared on land not previously grown to tomato and not known to have Fusarium wilt. The pathogen-infested field had been previously established over several (~5) years of planting Fol R3-inoculated tomatoes and incorporating infested plant tissue at the end of each season. In the year prior to the study, the incidence of Fusarium wilt was ~50%–80% across the whole field, and no other soil-borne diseases were detected. This field had a history of low tomato spotted wilt virus (TSWV) levels (leafhopper vectored), which we tested for in each year.

Transplants were produced by double seeding in 72-cell trays filled with a potting medium developed by Dina St. Clair (DAS) at UC Davis, 1 m^3^ of which consists of 22% perlite (*v*/*v*), 11% vermiculite, 26% sphagnum peat moss, 8% sand, and 10% composted redwood bark, with the remaining 24% made up of 2.03 kg Dolomite #65 AG, 2.25 kg APEX 14-14-14 (3–4 months) slow-release fertilizer, and 0.68 kg Micromax micronutrient fertilizer. The seeds were treated with ETOH and 1% sodium hypochlorite, as described above, prior to planting. Seedlings grew in the greenhouse for 5 weeks (fertilized weekly with a low-nutrient solution) and were hardened in an outdoor lath house for 1 week prior to planting. The plants designated for the infested field were dip inoculated with Fol R3 the day before transplant. To dip inoculate, whole 72-cell transplant trays were submerged up to the plant crown in ~20 L of a Fol R3 (isolate CS3) spore suspension of 10^6^ spores/ml 0.1% water agar (prepared as described above), allowed to absorb inoculum for 2 min, left to drain overnight, and then planted in 150-cm-wide beds at 30- to 35-cm spacing between plants. Plants were irrigated with subsurface drip buried ~10 cm below the soil line.

In 2019 only, the beds were treated with a pre-plant herbicide (Matrix; active ingredient, rimsulfuron) at the label rate 1 week prior to planting; mid-season, the plants were treated with imidacloprid (Advise) at the label rate to control whiteflies. In both years, fertigation applications with a starter fertilizer were performed at a rate of 30–33 L/ha over 4 weeks, starting in late May. Urea ammonium nitrate (UAN) solution at respective rates of 32, 12, and 14 L/ha were applied for 5 weeks starting the first week of July. To keep up with potassium requirements later in the season, sulfur/potassium thiosulfate (KTS 0-0-25) was applied once at a rate of 22.7 L/ha. To account for any nutrient-related plant health issues in 2019, the nutrient composition was analyzed in August in both fields based on two composite soil cores (five 10 cm × 1 cm cores) randomly collected from each of the two rows. Based on this analysis, we verified that the nutrient levels were optimal for tomato production in both fields, apart from the poor performing edge in the non-infested field (**Table 2**). The nitrogen levels were higher in the infested field compared to the non-infested field, but the two fields were not otherwise notably different.

**Table 2:** Field soil nutrient analyses for both the infested and non-infested fields at the UC Davis Plant Pathology Research Station (collected in 2019 only).

Field	pH	EC (ppm)	Nitrate nitrogen (N) (ppm)	Ammonium nitrogen (N) (ppm)	Phosphorus (P) (ppm)	Potassium (K) (ppm)	Calcium (Ca) (ppm)	Magnesium (Mg) (ppm)
Non-infested^a^	7.2	0.3	8.5	3	11.5	225	2,150	1,425
Infested	7.1	0.5	13	3	10	185	1,900	1,510

a Values in the non-infested field were averaged between poor-performing and well-performing border rows. Actual field values were estimated to fall within the range of the two.

##### Disease development and pathogen confirmation

Disease incidence was quantified in 10 plants per plot every other week starting from mid-June until harvest (September). Early symptoms included one-sided leaf chlorosis (**Figure 1D**) and plant stunting. These symptoms progressed into severe yellow flagging of whole branches and total plant collapse by harvest. At harvest, all plants were ranked as either healthy or with acute wilt-like symptoms, and vascular discoloration was evaluated in five plants per plot (**Figure 1E**).

Fungal tissue isolations were conducted from all symptomatic plants from each cultivar in order to establish whether the symptoms were due to Fol R3. A 10-cm section of the stem of each plant was washed in 0.1% Tween 20, dipped for 30 s in 70% ethanol and 2 min in 1% sodium hypochlorite, and then cut into 1-cm segments and placed onto *Fusarium* selective medium (FSM) in 100 mm agar Petri plates (as described in Swett et al., 2016). After 7–10 days, any *F. oxysporum*-like colonies were sub-cultured to a water agar medium amended with 0.5% KCl, as described by Swett et al. (2016). After 3–5 days of growth, these colonies were tentatively identified as *F. oxysporum* based on microscopic features, i.e., the presence of fusiform macroconidia and short, unbranched monophialids carrying false heads of microconidia, as described in Leslie and Summerell (2008). For cultures that were morphologically consistent with *F. oxysporum*, fungal tissue was prepared by growing 7 days on PDA (24°CC, 24:0 h L/D) and the DNA extracted using the PrepMan Ultra Sample Preparation kit (Applied Biosystems, Foster City, CA, USA) according to the manufacturer’s instructions, producing 100 μl of eluted DNA, which was stored at −20°CC. Diagnostic PCR of Fol for the *SIX3* gene region was conducted using the *SIX3* primers—SIX3-F1 (CCAGCCAGAAGGCCAGTTT) and SIX3-R2 (GGCAATTAACCACTCTGCC) (Lievens et al., 2008; Van Der Does et al., 2008)—in a T100 Bio-Rad thermal cycler (Hercules, CA, USA). Based on this, we confirmed Fusarium wilt in 20% of putative Fusarium wilt-affected plants in both 2018 and 2019.

Data were only included for plants in which Fol was confirmed within the sub-plot. The area under the disease progress curve (AUDPC) was calculated as described in Shaner and Finney (1977) using the last three data points. AUDPC was based on the percentage of plants with aboveground Fusarium wilt symptoms in each of the three blocks, while vascular discoloration incidence was calculated as the percentage of 10 plants in each block with vascular symptoms at harvest (*n* = 3 for each year, *n* = 6 for the two years combined). As described in detail above, based on the estimated off-type rate of 2% of seeds, disease development in >2% of plants was considered indicative of ineffective *I3* gene expression.

#### Greenhouse-based evaluation of Fol R3 resistance durability and development of a robust phenotyping method for Fol race 4 identification

All trials were conducted with three plants per cultivar per isolate and included one cultivar each that was: 1) susceptible to all races (EP 7 or Brandywine); 2) susceptible to race 2 and race 3 only (H 9036); 3) susceptible to race 3 only (H 8504); and 4) three cultivars resistant to race 3 (HM 5235, HM 58801, and N 6428) (**Table 1**). Pathogen treatments consisted of the Fol R3 isolate CS3 and the mock-inoculated negative controls (0.1% sterile water agar). The experiment was arranged by isolate treatment to avoid cross-contamination. The study was conducted three times.

Tomato seeds were treated as described above and then planted into Corlite soil in 50-well seedling trays with 0.5 g of osmocote slow-release fertilizer on top of each cell. The plants were watered daily as needed and transplanted into 3.8-L pots with Corlite soil 2 weeks post-germination. One week after transplanting, the plants were inoculated with a spore suspension prepared from ten 7-day-old cultures grown on PDA (24°CC, 24-h light). This was first suspended in a total of 100 ml sterile 0.5% KCl, and then an additional 600 ml sterile 0.1% water agar was added for a total volume of 700 ml. A 50-ml inoculum was added to each plant. Mock-inoculated plants were treated with 50 ml 0.1% water agar. The plants were watered by hand for 2 weeks to ensure the inoculum was not washed out of the pots and were then placed on automatic drip irrigation, wherein water and fertilizer were applied daily, adjusted to plant needs. Foliar symptoms of wilt and chlorosis and stem vascular discoloration were evaluated 60 days post-inoculation. Disease development was quantified based on the percentage of plants across the three repeat experiments (*n* = 3) that developed vascular discoloration and wilt/severe chlorosis. As described in detail above, based on the estimated off-type rate of 2% of seeds, disease development in >2% of plants was considered indicative of ineffective *I3* gene expression. The presence of symptoms in non-inoculated controls was used to determine the presence of potential contaminant pathogens: should symptoms have been observed, fungal isolation from tissue would have been conducted to determine the identity of the pathogen contaminants. However, symptoms were never observed in the non-inoculated controls.

### Statewide survey for Fol race 4 in commercial F3 processing tomato fields over 5 years

Statewide surveys for Fol race 4 in California processing tomato production were conducted annually from 2017 through 2021 in F3 (Fol race 3-resistant) cultivar fields that displayed Fusarium wilt-like symptoms. Fields were evaluated in Fresno, Merced, San Joaquin, Yolo, Colusa, and the Sutter counties, the latter county being the putative site of origin for Fol R3 in the state (Cai et al., 2003). Three to five plants were collected from each field for diagnosis. The diagnosis consisted of symptom photodocumentation, followed by fungal isolation from the diseased stem tissue and tentative morphological identification as described above. To determine whether the *F. oxysporum* isolates were putatively Fol, *SIX3* PCR diagnosis was conducted with two to five *F. oxysporum* isolates per field (Lievens et al., 2008) as described above.

All putative Fol isolates were first purified with a single hyphal tip and stored on filter paper at 4°CC. To confirm that the pure cultured isolate retained the same putative Fol identity as the original non-purified isolate (which may have represented a mixed culture), genomic DNA was extracted from tissue as above, but using the Quick-DNA Fecal/Soil Microbe Miniprep Kit (Zymo, Irvine, CA, USA) according to the manufacturer’s instructions, producing 100 μl of eluted DNA, which was stored at −20°CC. *SIX3* amplification was conducted with the genomic DNA. It should be noted that all isolates without *SIX3* amplification were further evaluated for *SIX1* amplification (as described in Lievens et al., 2008); however, these data were not presented as amplification of *SIX1* was not observed in all cases.

To confirm *F. oxysporum* identity, sequence-based species identity was analyzed for all isolates with *SIX3* amplification. An ~600-bp region of translation elongation factor 1-α (*tef1*) was amplified in a 30-μl volume using Platinum II Hot-Start PCR Master Mix (Invitrogen, Waltham, MA, USA) according to the manufacturer’s instructions using ef1 and ef2 primers (O’Donnell et al., 1998) in a T100 Bio-Rad thermal cycler (Hercules, CA, USA). The amplification conditions consisted of 94°CC for 5 min, 40 cycles of 94°CC for 45 s, 52°CC for 30 s, and 72°CC for 1.5 min, plus a final extension of 72°CC for 6 min. The PCR products were purified using PCR Product Cleanup Reagent ExoSAP-IT™ (Applied Biosystems, Waltham, MA, USA) according to the manufacturer’s instructions. The purified amplicons were sequenced with the same forward and reverse primers using automated sequencing at Eurofins Genomics (Louisville, KY, USA). BLAST analysis of the *tef1* sequences was conducted using both the Fusarium ID and GenBank databases. Isolates were identified at the species level based on the criteria of 98% or greater sequence homology. When ambiguous in GenBank, preference was given to the identity based on Fusarium ID due to the higher quality of identity assignments given to the reference sequences in this database.

Pure culture isolates with 98% or greater homology to *F. oxysporum* and amplification of the *SIX3* gene regions were further evaluated for subspecies identity using *in planta* phenotype analyses. One to two isolates per field were evaluated in greenhouse phenotyping trials using the potted plant method described above, with Fol, Forl, and mock-inoculated (negative) controls. To reduce ambiguities associated with disease development in F3 cultivars, three F3 cultivars (resistant to race 1, race 2, and race 3) were included: HM 5325, HM 58801, and N 6428. Based on initial protocol development studies (described further below in *Results*), an isolate was considered Fol race 4 if it could cause wilt and vascular discoloration in at least one of three plants for all F3 cultivars and the Forl-resistant cultivar (HMX 4909) in order to exclude the possibility that the isolate was Forl (despite positive *SIX3* PCR diagnosis).

## Results

### Accuracy of different *in planta* tools using differential cultivar resistance for formae specialis and race differentiation, which varied in testing duration

#### Lab bio assay

Pathogen treatment had a significant effect on lesion development (*p* < 0.01), but cultivar treatment did not (*p* = 0.27). Overall, fewer mock-inoculated seedlings developed radicle and hypocotyl lesions (3%–20%) than seedlings in any of the *F. oxysporum* treatments (*p* < 0.01). Seedlings inoculated with all the pathogen treatments (Fol R3 and Forl), as well as the nonpathogenic isolate (Fonp), developed large brown lesions on the hypocotyl and radicle. The percentage of seedlings with lesions was not significantly different (*p* = 0.24) between Fol R3, Forl, and Fonp, wherein 40%–88% of seedlings developed lesions across all *F. oxysporum* × cultivar treatment combinations (**Table 3**).

**Table 3:** Phenotyping with a rapid seedling laboratory bioassay of four tomato cultivars inoculated with different formae specialis *Fusarium oxysporum* strains.

Pathogen^a^	Cultivar^b^	Symptomatic seedling (%)^c^	Seedling mortality (%)^c^
Fol race 3	H 8504 (F2)	74.3 ± 10.92 b	15.9 ± 11.21 b
	HM 58841 (F2)	78.3 ± 21.62 b	39.7 ± 28.06 b
	H 1310 (F3)	86.9 ± 1.69 b	24.7 ± 14.56 b
	HMX 4909 (Fr)	43.4 ± 3.37 b	7.8 ± 5.55 a
Forl	H 8504 (F2)	74.9 ± 8.20 b	49.2 ± 34.82 b
	HM 58841 (F2)	88.3 ± 6.87 b	32.1 ± 22.69 b
	H 1310 (F3)	89.4 ± 10.55 b	60.2 ± 42.59 b
	HMX 4909 (Fr)	50.4 ± 3.75 b	7.1 ± 5.0 a
Fonp	H 8504 (F2)	64.2 ± 20.0 b	36.0 ± 25.46 b
	HM 58841 (F2)	68.3 ± 31.66 b	54.8 ± 38.75 b
	H 1310 (F3)	57.5 ± 29.94 b	20.0 ± 14.16 b
	HMX 4909 (Fr)	32.3 ± 5.61 ab	10.1 ± 7.14
Water	H 8504 (F2)	29.6 ± 4.62 a	0.0 ± 0 a
	HM 58841 (F2)	11.4 ± 4.41 a	7.8 ± 5.55 a
	H 1310 (F3)	36.3 ± 26.34 a	0.0 ± 0 a
	HMX 4909 (Fr)	3.3 ± 3.33 a	2.3 ± 1.66 a
*p*-value pathogen treatment		<0.01	<0.01
*p-*value cultivar treatment		0.27	0.39
Pathogen × cultivar treatment		0.24	0.48

a Pathogen treatment: Fusarium oxysporum f. sp. lycopersici (Fol) race 3, Fusarium oxysporum radicis lycopersici (Forl), nonpathogenic F. oxysporum (Fonp), and water (mock-inoculated).

b Resistance abbreviations used in text: F1, resistant to Fol race 1 only; F2, resistant to Fol race 2; F3, resistant to Fol race 3; Fr, resistant to Forl.

c Symptomatic seedlings (percentage) consisted of seedlings with radicle and hypocotyl lesions. Radicle and hypocotyl symptoms and seedling mortality were evaluated 6 days after inoculation. Values in a column followed by the same letter were not significantly different according to the least square means significant difference (p < 0.05). Plus-minus values represent the standard error of the mean. Variables were analyzed using a type II test with the R “car” package.

Similarly, pathogen treatment had a significant effect (*p* < 0.01) on seedling death, but cultivar treatment did not (*p* = 0.39). Fewer mock-inoculated seedlings died (0%–7% mortality) than in any of the *F. oxysporum* treatments (*p* = 0.005). Seedling mortality did not significantly vary between Fol R3, Forl, and Fonp, which ranged from 40% to 88% across all cultivars tested (*p* = 0.99) (**Table 3**).

#### Young plant tray assay

Plants inoculated with Fonp and the mock-inoculated (untreated) plants did not develop any symptoms throughout the experiment. These treatments were thus excluded from downstream statistical analyses.

##### Fol race 3

Cultivar had a significant effect (*p* < 0.01) on the percentage of plants with vascular discoloration, wherein all Fol R3-suceptible cultivars (i.e., H 8504, H 9036, HM 58841, HMX 4909, and Brandywine) developed vascular discoloration in 31%–78% of plants. The F3 cultivars (i.e., H 1310 and N 6428) did not develop vascular discoloration (**Table 4**). Plant decline was variable across experiments, wherein only Brandywine plants wilted in experiment 1 (15% of plants), while wilt developed in 4%–19% of the plants in all Fol R3-suceptible cultivar treatments in experiment 2. Plant decline was never observed in the F3 cultivars (H 1310 and N 6428).

**Table 4:** Phenotyping with a young plant tray assay of seven tomato cultivars inoculated with different formae specialis of *Fusarium oxysporum*.

Fol race 3^a^				Forl^a^	
Cultivar^b^	Vascular discoloration (%)^c^	Plant decline (%) exp. 1^c^	Plant decline (%) exp. 2^c^	Crown rot (%)^c^	Plant decline (%)^c^
H 8504 (F2)	31.9 ± 11.88 b	0.0	17.7	95.8 ± 4.16 b	7.3 ± 1.04 a
Brandywine (none)	78.6 ± 21.42 b	15.4	19.4	90.6 ± 9.37 b	9.4 ± 3.12 a
H 9036 (F1)	38.9 ± 5.5 b	0.0	15.7	96.9 ± 3.12 b	18.7 ± 12.5 a
H 1310 (F3)	0.0 ± 0 a	0.0	0.0	92.8 ± 7.14 b	10.5 ± 3.80 a
N 6428 (F3)	0.0 ± 0 a	0.0	0.0	86.6 ± 6.66 b	10.0 ± 10.0 a
HM 58841 (F2)	36.6 ± 0.89 b	0.0	4.4	90.0 ± 1.00 b	19.4 ± 0.62 a
HMX 4909 (Fr)	46.9 ± 3.12 b	0.0	8.8	0.0 ± 0 a	0.0 ± 0 a
*p*-value pathogen treatment	*<0.01*	*NA*	*NA*	*<0.01*	*NA*
*p*-value cultivar treatment	*<0.01*	*NA*	*NA*	*<0.01*	*0.257*
Pathogen × cultivar treatment	*<0.01*	*NA*	*NA*	*<0.01*	*NA*

NA: statistical analyses were not performed as the plant mortality incidence was variable among the experiment replicates.

a Pathogen treatment: Fusarium oxysporum f. sp. lycopersici (Fol) race 3 and Fusarium oxysporum radicis lycopersici (Forl). The nonpathogenic F. oxysporum (Fonp) and mock-inoculated treatments did not develop symptoms and were not included in the analyses.

b Resistance abbreviation used in text: No resistance, no Fol or Forl resistance; F1, resistant to Fol race 1 only; F2, resistant to Fol race 2; F3, resistant to Fol race 3; Fr, resistant to Forl.

c Values in a column followed by the same letter were not significantly different according to the least square means significant difference (p < 0.05). Plus-minus values denote standard error of the mean. Variables were analyzed using a type II test with the “car” package in R. Symptoms were evaluated 7 weeks after pathogen inoculation.

##### Forl

Cultivar had a significant effect (*p* < 0.01) on the percentage of seedlings with crown rot. While the Forl cultivar (HMX 4909) never developed crown rot, at least 86% of the Forl-susceptible cultivars developed crown rot. Plant decline was minor in all trials (7%–19% of plants) and did not vary by cultivar (*p* = 0.25), likely reflecting abiotic stresses in the high-density tray environment. No plants died in any treatment (**Table 4**).

#### Young plant single-pot assay

Plants inoculated with Fonp and the mock-inoculated (0.1% water agar) plants did not develop any symptoms throughout the experiment. These treatments were therefore excluded from subsequent statistical analyses.

##### Fol race 3

Cultivar had a significant effect (*p* = 0.04) on the percentage of plants with vascular discoloration. Across the Fol R3-susceptible cultivars (i.e., HM 58841, HMX 4909, and H 8504), 41%–100% of plants developed vascular discoloration, whereas one plant in the Fol R3-resistant cultivar H 1310 developed vascular discoloration (an issue further addressed in the Fol R3–F3 interaction studies) (**Table 5**). Plant decline only developed in the susceptible cultivars H 8504 and HMX 4909, in less than 8% of plants.

**Table 5:** Phenotyping with a young plant single-pot plant assay of four tomato cultivars inoculated with different formae specialis of *Fusarium oxysporum*.

	Fol race 3^a^		Forl^a^
Cultivar^b^	Vascular discoloration (%)^c^	Plant decline (%)^c^	Crown rot (%)
H 8504 (F2)	100.0 ± 0 b	8.33 ± 8.3 a	100 ± 0.0 b
HM 58841 (F2)	41.6 ± 8.3 ab	0.0 ± 0 a	100 ± 0.0 b
H 1310 (F3)	8.3 ± 8.3 a	0.0 ± 0 a	91.6 ± 8.33 b
HMX 4909 (Fr)	66.6 ± 16.6 b	8.33 ± 8.3 a	0.0 ± 0 a
*p*-value pathogen treatment	*<0.01*	NA	*<0.01*
*p*-value cultivar treatment	*0.049*	*0.615*	*<0.01*
Pathogen × cultivar treatment	*<0.01*	NA	*<0.01*

NA, not applicable.

a Pathogen treatment: Fusarium oxysporum f. sp. lycopersici (Fol) race 3 and Fusarium oxysporum radicis lycopersici (Forl). The nonpathogenic F. oxysporum (Fonp) and mock-inoculated treatments did not develop symptoms and were not included in the analyses.

b Resistance abbreviations used in text: F2, resistant to Fol race 2; F3, resistant to Fol race 3; Fr, resistant to Forl.

c Values in a column followed by the same letter were not significantly different according to the least square means significant difference (p < 0.05). Plus-minus values denote the standard error of the mean. Variables were analyzed using a type II test with the “car” package in R.

##### Forl

Cultivar had a significant effect (*p* < 0.01) on the percentage of plants with crown rot symptoms. The Forl-resistant cultivar HMX 4909 never developed crown rot symptoms. In contrast, crown rot developed in at least 91% of the plants across all Forl-susceptible cultivars (i.e., HMX 58801, H 8504, and H 1310). Plant decline was not observed in any treatment.

### Evaluating the relationship between Fol R3 and F3 processing tomato cultivars to overcome the challenges of *in planta* diagnostics

#### Field-based evaluation of the relationship between the Fol R3 and F3 cultivars

##### Foliar symptom development

Across years, chlorosis and wilt symptoms developed in 59.4% ± 3.5% of the Fol R3-suceptible positive control plants (i.e., H 8504 cultivar) in the infested field. Chlorosis and wilt symptoms also developed in two of the three Fol R3-resistant processing tomato cultivars, H 1310 and N 6428, with the greatest AUDPC in H 1310. HM 58801 never developed wilt symptoms (**Table 6**).

**Table 6:** Fusarium wilt development in Fol race 3-resistant (F3) cultivars grown in a field artificially infested with Fol race 3 (2018 and 2019).

Vascular discoloration^a^				AUDPC^b^
Cultivar^c^	2018	2019	Exp combined	Exp combined
H 1310	0% ± 0%	36.7% ± 17.6%	18.3% ± 11.4%	25.67 ± 8.56
N 6428	0% ± 0%	10% ± 6%	3% ± 3%	3.5 ± 3.5
HM 58801	0% ± 0%	3% ± 3%	1.6% ± 1.6%	0 ± 0

a Incidence was calculated as the percentage of plants positive for Fusarium wilt (out of 10) based on confirmed vascular discoloration and positive SIX3 PCR.

b AUDPC (area under the disease progress curve) was calculated as a measure of disease progression throughout the season for both years. Calculations were based on wilt incidence measurements collected at 2-week intervals starting 2 weeks after transplant and continuing until harvest. N = 20 for the 2018 and 2019 trials combined.

c H 1310, N 6428, and HM 58801 are all F3 cultivars with single-gene (I3 gene) quantitative resistance to Fol race 3. The positive F2 control H 8504 developed vascular discoloration in 87% ± 7% of plants across both years.

##### Development of vascular discoloration

The positive F2 control H 8504 developed vascular discoloration in 87% ± 7% of plants across both years. Vascular discoloration was detected in 2019 in all three F3 cultivars, although it was only detected in <2% of plants (accounting for standard error) in H 1310 (37% of plants) and N6428 (10% of plants) (**Table 6**). With the exception of a low level of TSWV, which was detected and rouged out of plots early in the study, mock-inoculated plants did not develop symptoms of other biotic or abiotic disorders, indicating that Fol R3 was the sole driver of wilt and vascular discoloration symptoms.

#### Greenhouse-based evaluation of Fol R3 resistance durability and development of a robust phenotyping method for Fol race 4 identification

All Fol R3-suceptible cultivars (i.e., H 9036, HMX 4909, H 8504, and Brandywine) developed both wilting/severe chlorosis (56%–100% of plants across cultivars) and vascular discoloration (44%–89% of plants across cultivars). Two Fol R3-resistant cultivars, HM 5235 and HM 58801, never developed vascular discoloration, although 11% of HM 58801 plants developed wilt/severe chlorosis. The Fol R3-resistant cultivar N 6428 developed both wilt and vascular discoloration in one trial (33% of plants), with an average of 11% of plants symptomatic across all studies. In all trials, mock-inoculated plants developed no symptoms (**Table 7**).

**Table 7:** Fusarium wilt development in resistant (F3) cultivars under greenhouse conditions.

Fol race 3 resistance status	Cultivars (resistance)^a^	Severe chlorosis/wilting (%)^b^	Stem necrosis/vascular discoloration (%)^b^
Resistant	HM 5235 (F3)	0% ± 0%	0% ± 0%
	HM 58801 (F3)	11% ± 11%	0% ± 0%
	N 6428 (F3)	11% ± 11%	11% ± 11%
Susceptible	H 9036 (F1)	67% ± 19%	67% ± 19%
	HMX 4909 (F2, Fr)	56% ± 11%	44% ± 11%
	H 8504 (F2)	100% ± 0%	89% ± 11%
	Brandywine (no R)	89% ± 11%	56% ± 11%

a Cultivar resistance status: F3 = resistant to Fusarium oxysporum f. sp. lycopersici (Fol) race 3; F2 = resistant to Fol race 2; F1 = resistant to Fol race 1; Fr = resistant to Fusarium oxysporum f. sp. racidis lycopersici (Forl); no R = no Fol or Forl resistance.

b Mean of three repeat experiments based on the percentage of plants (n = 3). Plus-minus values denote the standard error of the mean.

### Statewide survey for Fol race 4 in commercial F3 processing tomato fields over 5 years

Overall, in evaluations of putative Fol isolates from 17 F3 processing tomato fields over 5 years, Fol race 4 was never detected (**Table 8**). Based on the phenotyping of pure cultured, *SIX3*-positive *F. oxysporum* isolates, every isolate was identified as Fol R3. The Fol race 1 and race 2 isolate positive controls included in this had expected phenotypes, which confirmed that the race identification was accurate. Of note is that, in 2018, 2019, and 2020, 15%–100% of the isolates later identified as Fonp or Forl were positive for the *SIX* gene region. However, in repeat *SIX* analysis of the pure cultured isolates used in phenotyping, these isolates were all negative for the *SIX3* gene region. It is possible that these represent cases where Fol was originally present in mixed culture with Forl or a nonpathogenic strain, but were lost in the pure culturing process.

**Table 8:** Outcome of *Fusarium oxysporum* f. sp. *lycopersici* (Fol) race 4 statewide monitoring in California processing tomatoes over 5 years: from 2017 to 2021.

Year	Total	No. of fields (%)^a^					
		Fol				Forl	Non-Path
		R1	R2	R3	R4		
2017	2	0	0	2 (100%)	0	0	0
2018	11	0	0	11 (100%)	0	0	0
2019	0	0	0	0	0	0	0
2020	2	0	0	2 (100%)	0	0	0
2021	2	0	0	2 (100%)	0	0	0
Total	17	0	0	17 (71%)	0	0	0

a Number (and percentage) of fields for which F. oxysporum SIX3 PCR-positive isolates were tested and number (and percentage) identified as Fusarium oxysporum f. sp. racidis lycopersici (Forl), Fol races (1–4), and as nonpathogenic (Non-Path) F. oxysporum. Of note is that all isolates without SIX3 amplification were further evaluated for SIX1 amplification; however, in all cases, SIX1 amplification was not observed.

## Discussion

Fol race 4 has, thus far, gone unreported worldwide. The first detection of an *I3*-resistance-breaking Fol race 4 will be the signal to the global breeding industry to start developing race 4 resistance. Fusarium wilt resistance in California processing tomatoes has a history of remaining effective for up to 12 years (Walker, 1971; Zobel, 1971; Thomas, 1979; Miyao, 1980; Miyao and Debiase, 1996; Cai et al., 2003; PTAB, 2016). It has been 13 years since the first F3 materials were planted commercially in California (2009), but only 5 years since their widespread use (2016)—a timeline that indicates the imminence of the emergence of Fol race 4. Once a new race is detected, it typically has taken at least 10 years for resistance development and commercial adoption. Containment to the area(s) of detection will therefore be critical to mitigating the impact, a rapid response that in turn requires both rapid and accurate diagnosis.

Due to the challenges of the field-based and the morphology- and molecular-based diagnosis described above, the most promising approach for Fol race 4 monitoring would be the combination of tentative morphology and Fol PCR diagnostics with phenotyping using differential cultivars. Our efforts to identify and validate an accurate and efficient *in planta* tool for Fol race 4 monitoring indicated that rapid seedling-based methods will not be useful as they cannot differentiate between the pathogenic and nonpathogenic *F. oxysporum* strains or between Fol and Forl, nor can they reliably differentiate a susceptible from a resistant cultivar response. The ability of Fonp to cause lesions on the germinating hypocotyls and of Fol and Forl to cause disease on resistant seedlings likely reflects the extreme susceptibility of the seedlings grown under these conditions.

Transitioning to young plant greenhouse-based phenotyping methods has removed the false positives for Fonp and allowed reliable differentiation between Fol and Forl using susceptible/resistant differentials. It was possible to use a high-throughput (tray-based) approach to obtain results 7 weeks after inoculation. However, the development of severe foliar decline symptoms in resistant cultivars under the high-stress conditions in the tray system could pose challenges to the interpretation of the trials; this method also required larger inoculum volumes, which can be prohibitory in screening large isolate numbers.

While the single-pot method took the longest for symptom development (11 weeks), it was the easiest to use for large numbers of isolates (inoculum preparation was simple), was consistent and accurate in differentiating among formae specialis, and never resulted in severe foliar symptoms in resistant cultivars. This, therefore, represented the most reliable method for the detection of Fol race 4, and it was the method we utilized for downstream race 4 screening. However, it is concerning that the long duration required for this method is not conducive to a rapid response to Fol race 4 detection, which increases the chances of Fol race 4 dispersal beyond the initial detection site before barriers can be placed for containment.

Further complicating the *in planta* phenotyping efforts (as well as field diagnosis in support of race 4 monitoring efforts), early observations indicated that Fol R3 may be able to cause disease in cultivars with the *I3* resistance gene (F3 cultivars), and this ability was confirmed in both field and greenhouse studies. Disease incidences of as high as 30% in the greenhouse and field trials suggested that this is not solely an issue of non-hybridized (*I3* absent) individuals developing symptoms, but rather points to the potential inefficacy of *I3* gene expression in processing tomatoes—a phenomenon not observed in fresh market materials (G. Vallad, personal communication). While this phenomenon is not, to the authors’ knowledge, previously described for the *I3* gene in tomato, issues of inconsistent resistance gene expression have been documented for other Fol resistance genes. Alon et al. explored this as early as 1973, with their study evaluating incomplete penetrance of the tomato cultivars with the *I1* resistance gene. In this study, the *I1* resistance gene was incompletely expressed under high inoculum levels and at lower soil temperatures (Alon et al., 1974). It is possible that abiotic and biotic stressors compromise the expression of the *I3* resistance gene, a possibility supported by studies on soil salinity (Swett, 2020). For the purposes of monitoring race 4 emergence, standard phenotyping methods may generate false positives for race 4. However, our studies suggest that the inclusion of multiple F3 cultivars can overcome this challenge. Inconsistencies in the expression of the *I3* gene in processing tomatoes are going to be critical to understand and overcome both for improved field management of Fusarium wilt and for more effective monitoring of race 4.

For the purposes of Fol race 4 detection, greenhouse studies indicated that a three-F3 cultivar system can overcome the challenges posed by Fol R3 wilt development in F3 cultivars, using the parameter that all three F3 cultivars must develop disease in order to be considered Fol race 4. Using this method, we detected putative Fol in 17 F3 fields expressing Fusarium wilt-like disease symptoms during Fol race 4 monitoring efforts over 5 years. This method was consistently able to accurately and non-ambiguously identify associated *F. oxysporum* strains.

Of the 17 Fol detections in F3 fields, Fol race 4 was never recovered. At present, a large portion of the state is still utilizing susceptible (F2) cultivars, preventing thorough monitoring. However, the area affected by Fol R3 is much greater than that of the previous races: with larger population sizes, there is an increased chance of a random mutation resulting in resistance breaking. It was notable that there were an additional six F3 fields that were initially identified as Fol based on *SIX3* amplification, but the subsequent pure culture was not *SIX*-positive. These might have represented cases of mixed infections with Fol and non-Fol *F. oxysporum* isolates, wherein the Fol isolate was lost. It was also notable that, while the *SIX* gene regions for Fol are known to generate false positives in the case of Fonp isolates (Jelinski et al., 2017), in this work, *SIX3* had valuable utility as a diagnostic tool for race 4 monitoring in California. Of all the nonpathogenic and Forl California isolates examined in this study, none were positive for the *SIX3* region. However, the possibility of false negatives (Fol that are negative for SIX) has not been examined. This should be more fully explored to determine whether the existing PCR diagnostic methods have a risk of misdiagnosing Fol race 4 as Forl or as a nonpathogen.

The need for the early and rapid detection of Fol race 4 is greater than that of the previous races, given the changes in machine use practices in the last two decades. When Fol R3 emerged, most growers in the state were still harvesting their own tomatoes. In contrast, the majority of acreage in the state is currently custom harvested by canneries using the same harvesters, trailers, and tractors in fields across the state. The importance of equipment-based spread is underscored by the recent statewide expansion of Fol R3 from the northern Central Valley into distant counties such as Fresno (2014) and Kern (2018) following these changes in production practice (Swett, unpublished data).

A realistic management goal for resistance-breaking Fol race 4 is not to completely eradicate the pathogen from the field, nor to completely prevent spread, but instead to reduce the population loads in the field and slow dispersal in order to provide the breeding industry time to identify new resistance and integrate into commercial materials. The management of soil-borne pathogen spread on shared equipment is currently being evaluated in synergistic efforts to develop the best sanitation practices for shared equipment, as no methods currently exist and these practices are challenging to implement for harvesters and other high-risk equipment. Chemical treatment strategies may be effective in reducing the Fol race 4 populations in affected sites (Aegerter and Swett, 2021; Paugh et al., 2022, in press). In addition, the use of crop rotations, which are poor asymptomatic hosts (such as grass crops), and avoidance of cryptic systemic hosts (such as melons) may be important to mitigate population buildup in the field (Paugh and Swett, 2021).

As a further consideration, a broad-spectrum resistance gene for all Fol races has been described in tomato (e.g., the *I7* gene) (Chitwood-Brown et al., 2021a). If the breeding industry proactively incorporated broad-spectrum resistance into existing processing tomato materials, this could significantly extend the efficacy of Fusarium wilt resistance as a management tool.

Monitoring for Fusarium wilt resistance breaking is a priority in dozens of different crops that use single-gene resistance to manage *F. oxysporum* formae specialis causing wilt diseases (Gordon and Martyn, 1997; Jiménez-Gasco et al., 2004; Diaz et al., 2021). While this study was focused on the California processing tomato production system, the lessons learned here regarding the need for standardized phenotyping methods and the inconsistencies in the durability of the resistance gene are applicable to resistance-breaking monitoring efforts not just for Fol in particular but also for *F. oxysporum* wilt pathogens in diverse crops worldwide.

## Data availability statement

The raw data supporting the conclusions of this article will be made available by the authors, without undue reservation.

## Author contributions

CLS: Lead manuscript author, conducted primary literature review, and analyzed data for F3/Fol R3 trials and Fol R4 surveys. JDCM: Coordinated, conducted, and analyzed data for phenotyping optimization studies; assisted in writing the introduction, discussion, and abstract; contributed to the literature review; and assisted in manuscript edits. EH: Coordinated, conducted, and analyzed data; wrote the methods for the F3/Fol R3 field trial; coordinated survey efforts; conducted diagnoses; curated isolates and conducted isolate phenotyping; and conducted F3/Fol R3 cultivar greenhouse trials. EHelp: Curated isolates, conducted isolate phenotyping, and contributed to writing methods for phenotyping trials. MG: Conducted diagnoses, curated isolates, conducted isolate phenotyping, and conducted F3/Fol R3 cultivar greenhouse trials. ELR: Conducted molecular analysis for curated isolates and isolate phenotyping. HJ: Conducted isolate phenotyping. RO, AH, and JB: Curated isolates and conducted isolate phenotyping. FR: Conducted molecular analysis for survey and curated isolates. All authors contributed to the article and approved the submitted version.
